# Does attachment removal damage enamel? Influence of adhesive, resin, and bur on surface integrity

**DOI:** 10.1111/eos.70088

**Published:** 2026-03-18

**Authors:** Marilia Daniela Busnardo Canadas, Ana Beatriz Silva Sousa, Fernanda de Carvalho Panzeri

**Affiliations:** ^1^ Department of Pediatric Dentistry Ribeirão Preto School of Dentistry University of São Paulo Ribeirão Preto Brazil; ^2^ Department of Restorative Dentistry University of Ribeirão Preto (UNAERP) Ribeirão Preto Brazil; ^3^ Department of Dental Materials and Prosthodontics Ribeirão Preto School of Dentistry University of São Paulo Ribeirão Preto Brazil

**Keywords:** adhesive systems, artificial aging, clear aligners, enamel roughness, orthodontic attachments, surface integrity

## Abstract

To evaluate the combination of composites, adhesive systems, and burs considering artificial accelerated aging (AAA), influence enamel roughness and morphology after attachment removal. One hundred ninety‐two bovine enamel specimens were divided according to composite type (Transbond XT, flowable, bulk‐fill flowable), adhesive system (Single Bond Universal or Transbond XT primer), and bur for attachment removal (multilaminated carbide or zirconia). Half of the samples were stored for 24 h at 37°C and the other half underwent 300 h of AAA. Surface roughness was measured using confocal laser scanning microscopy before and after attachment removal. Data were analyzed using three‐way anova with Bonferroni post hoc tests. Significant differences in enamel roughness were observed according to composite type, adhesive system, and bur used. Multilaminated bur produced higher surface roughness compared to zirconia bur. Transbond composite, particularly when combined with orthodontic adhesive, exhibited greater enamel alteration after AAA. None of the protocols fully restored enamel surface to its original smoothness. Attachment removal inevitably alters enamel morphology, regardless of the materials used. Composite, adhesive, and bur's choice, as well as proper finishing and polishing, is essential to minimize enamel damage after clear aligner treatment.

## INTRODUCTION

In recent years, orthodontics has undergone significant advances, driven by the increasing demand for esthetics and the growing popularity of clear aligners. These removable, transparent thermoplastic devices have become viable options for more complex treatments due to technological developments [1]. Compared to fixed appliances, aligners offer advantages such as enhanced esthetics, comfort, easier oral hygiene, and reduced pain [1, 2, 3, 4]. However, they present biomechanical limitations, particularly in complex tooth movements [5]. To overcome these challenges, small composite resin bulges—known as *attachments*—are bonded to the enamel to provide mechanical retention and facilitate three‐dimensional tooth control [1, 6, 7, 8].

The shape, size, and positioning of attachments are digitally planned using specialized software [9] and transferred intraorally with acetate templates filled with composite resin [10]. The choice of composite material directly affects clinical performance since viscosity and filler content influence adhesion, wear resistance, and dimensional stability [11]. Despite the widespread use of conventional, flowable, and bulk‐fill composites, few comparative studies have evaluated their performance in attachment build‐up [1, 7, 12, 13]. Furthermore, there is no consensus regarding the ideal material or viscosity, especially concerning enamel preservation during attachment removal, leaving the decision largely empirical [7, 14].

The adhesive system also plays a critical role in the bond strength between the tooth and attachments. The conventional orthodontic adhesives are unfilled [15], whereas the universal systems contain inorganic fillers and functional monomers that may enhance mechanical resistance [16]. However, while strong adhesion between enamel and composite resin ensures that the attachment remains stable throughout orthodontic treatment, preventing detachment during aligner use, this same bond stability becomes problematic during removal, increasing the risk of enamel damage [17, 18, 19, 20].

At the end of orthodontic therapy, the enamel surface should be restored to its original condition [21], minimizing roughness, demineralization, or biofilm retention [22]. Composite remnants are typically removed with multilaminated tungsten carbide burs [22, 23, 24] or zirconia burs [25], although the literature remains divided regarding their effects on enamel integrity. But, none of the available removal techniques completely preserves enamel structure [26, 27, 28]. Therefore, final polishing with rubber tips or abrasive pastes is essential to smooth the surface [28, 29, 30]. The restorative materials and removal protocol directly affect long‐term enamel health [31], underscoring the need to identify combinations that minimize surface damage, which is estimated to be around 5–20 µm of enamel loss [32].

Despite clinical advances, there is limited scientific evidence on the optimal combination of composites, adhesive systems, and removal techniques that best preserves enamel integrity. Therefore, this study aimed to evaluate which combination of adhesive system and composites is most suitable for attachment build‐up while maintaining enamel preservation after the removal at the end of the treatment.

The null hypotheses were: (1) enamel surface roughness does not differ after attachment removal regardless of the composite, adhesive system, or bur type used; and (2) no morphological alterations occur in the enamel structure after attachment removal regardless of the composite, adhesive system, or bur type used.

## MATERIAL AND METHODS

Sample size calculation (*n* = 8) was performed using OpenEpi software (www.openepi.com). A total of 192 enamel specimens (6 × 6 × 2 mm) were obtained from the buccal surfaces of bovine teeth. The enamel surfaces were flattened under water cooling using silicon carbide abrasive papers with progressively finer grits (400, 600, 1200, and 2000). Baseline surface roughness was measured using confocal laser scanning microscopy (VK‐X200, Keyence) at 20× magnification and a wavelength of 408 nm, with Sa standardized to 0.2 µm.

Confocal laser scanning microscopy enables high‐resolution imaging and topographic measurements with great depth of focus. The equipment combines conventional optical and laser microscopy (wavelength = 408 nm) to obtain images with excellent spatial resolution. Images are formed based on the light reflected by the sample, allowing identification of the highest and lowest points (*Z*‐axis) within the region of interest. Multiple optical slices are acquired at different Z‐positions and subsequently merged to generate a detailed three‐dimensional surface reconstruction.

The enamel surface of each specimen was 37% phosphoric acid etched (Condac 37%, FGM) for 30 s, rinsed with water for 30 s, and air‐dried for 15 s. Samples were randomly divided into two groups according to the adhesive system used: a conventional adhesive (Single Bond Universal, Solventum) or an orthodontic primer (Transbond XT, Solventum). The adhesive systems were actively applied following the manufacturer's instructions and light‐cured for 3 s at 3200 mW/cm^2^ (Valo, Ultradent) [33].

Attachments were 3.5 (width) × 1.0 mm (thickness), fabricated using a custom‐made acetate template to standardize position and volume on the buccal surface of the tooth, employing one of three composites: orthodontic composite (Transbond XT, Solventum), flowable composite (Filtek Supreme Flowable, Solventum), or bulk‐fill flowable composite (Filtek Bulk Fill Flow, Solventum). All composites were light‐cured for 3 s at 3200 mW/cm^2^ using the same curing unit (Valo, Ultradent) [34, 35].

After attachment preparation, half of the specimens were subjected to artificial accelerated aging (AAA) using a C‐UV nonmetallic accelerated weathering chamber (Comexim Matérias Primas Ltda).

AAA is commonly applied to evaluate the behavior of nonmetallic materials, exposing samples to visible light, UV radiation, alternating humid and dry conditions, and thermal cycling [36].

The exposure cycle consisted of 4 h of UV‐B irradiation (280–320 nm) at 50°C followed by 4 h of condensation at 50°C, for a total duration of 300 h, corresponding to approximately 1 year of clinical use [37]. The remaining samples were stored in artificial saliva at 37°C for 24 h [38].

**FIGURE 1:**
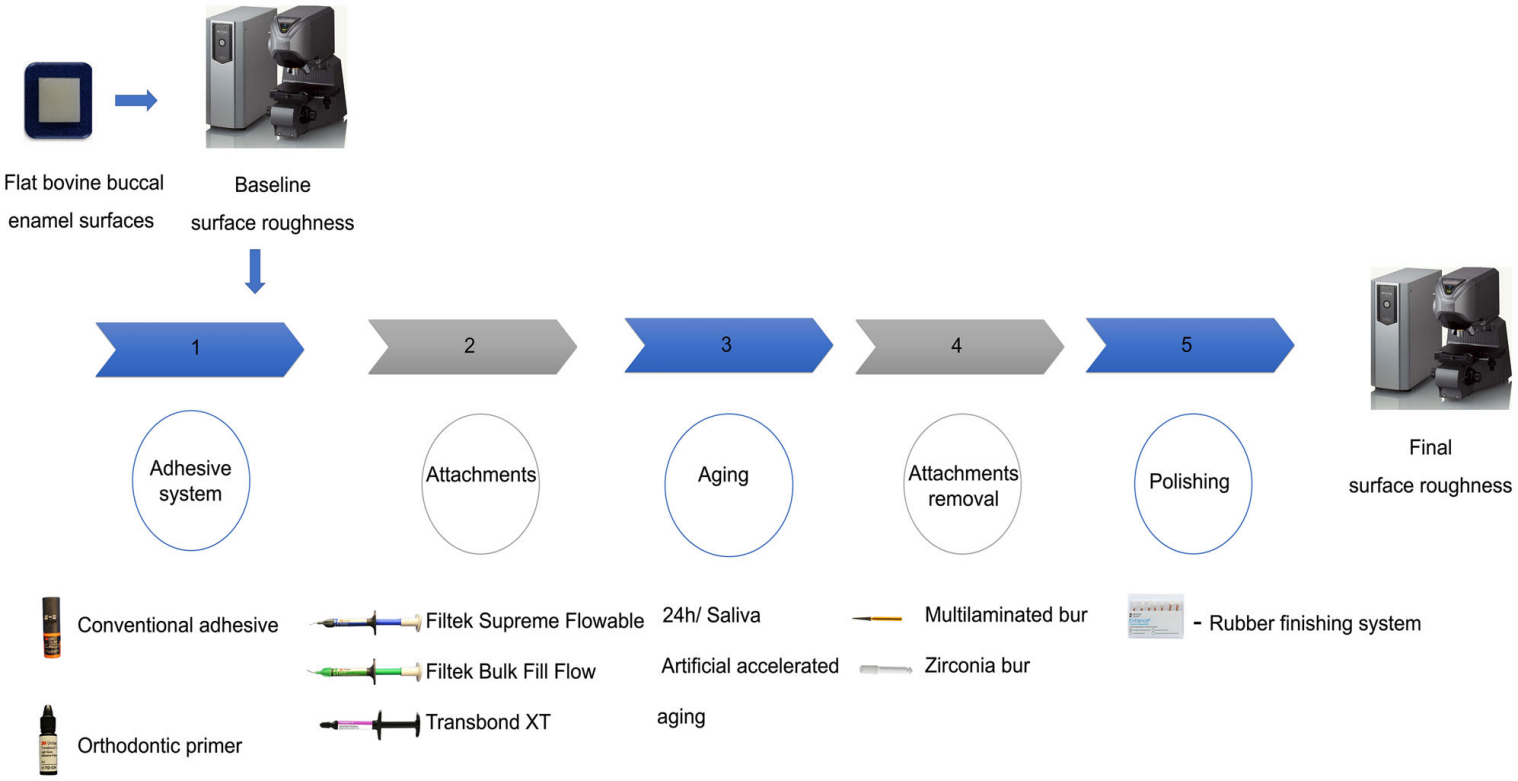
Flowchart of attachments building and experimental procedures.

Following aging, attachments were removed using either a 30‐blade multilaminated tungsten carbide bur (9114F, KG Sorensen) or a zirconia bur (Morelli). Complete composite removal was verified by visual inspection, and a new bur was used for every two samples. The enamel surfaces were polished using a rubber finishing system with flame‐shaped tips (Enhance, Dentsply Sirona). A single trained operator performed all experimental procedures to minimize interoperator variability. Surface roughness was then re‐evaluated using the same confocal laser scanning microscope and analyzed with VK Analyzer software (Keyence Corporation). Three‐dimensional surface reconstruction was performed at 1500× magnification using the same software (Figure 1).

All quantitative data were tested for normality using the Shapiro–Wilk test and for homogeneity of variance using Levene's test. Since the data were normally distributed, statistical analysis was performed using a three‐way anova followed by Bonferroni post hoc tests (*p* < 0.05).

## RESULTS

**FIGURE 2:**
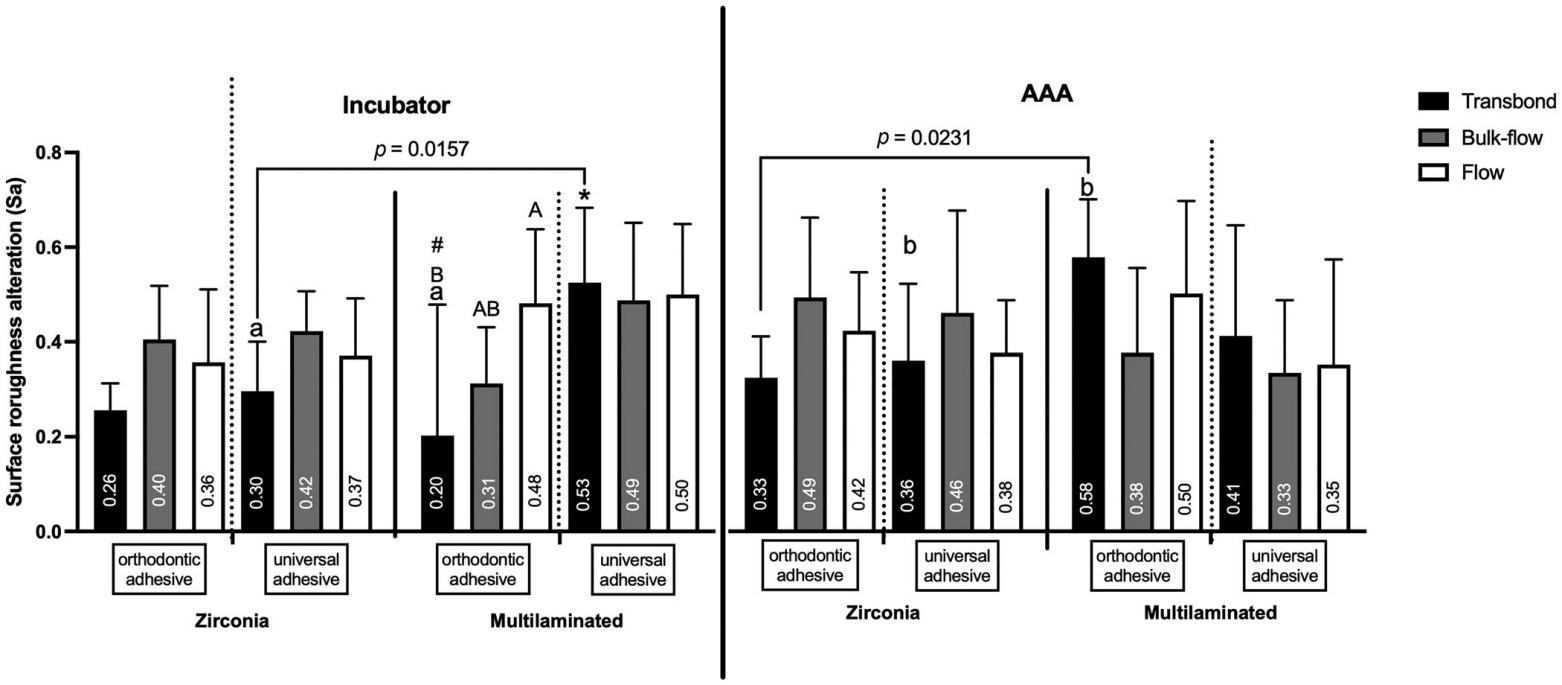
Comparison of the mean (±standard deviation) surface roughness changes after attachment removal and polishing across all variables (three‐way anova, Bonferroni post hoc test, *p* < 0.05). The numbers inside the columns indicate the mean Sa values. Different lowercase letters indicate statistically significant differences between aging conditions; different uppercase letters indicate statistically significant differences among the three composites within the same adhesive system; different symbols indicate statistically significant differences between adhesives for the same composite; and bars connected by lines represent statistically significant differences within the same composite and adhesive combination (*p* < 0.05). AAA, artificial accelerated aging.

The results of surface roughness alterations after attachment removal, considering the type of composite, adhesive system, bur, and aging condition, are illustrated in Figure 2.

When comparing mean surface roughness changes between samples using the same composite and adhesive system but different burs (zirconia vs. multilaminated carbide) under the incubator condition, a statistically significant difference (*p* < 0.05) was observed only for the Transbond composite combined with the universal adhesive when removed with the multilaminated bur.

For the orthodontic adhesive system, the use of Transbond composite also showed a statistically significant difference (*p *< 0.05) compared to the flowable composite when the multilaminated bur was used. Additionally, for the multilaminated bur, a significant difference (*p* < 0.05) was found between adhesive system types when used with the Transbond composite. No significant differences were detected in the other comparisons (*p* > 0.05).

Under AAA, a statistically significant difference (*p* < 0.05) was found only between bur types (zirconia vs. multilaminated) when the Transbond composite was used with the orthodontic adhesive system, with no significant differences observed among the remaining combinations (*p* > 0.05).

The artificially aged samples also showed significant differences when the Transbond composite and orthodontic adhesive system were used with the multilaminated bur, as well as when the same composite was used with the universal adhesive system and zirconia bur, both compared with the incubator condition.

**FIGURE 3:**
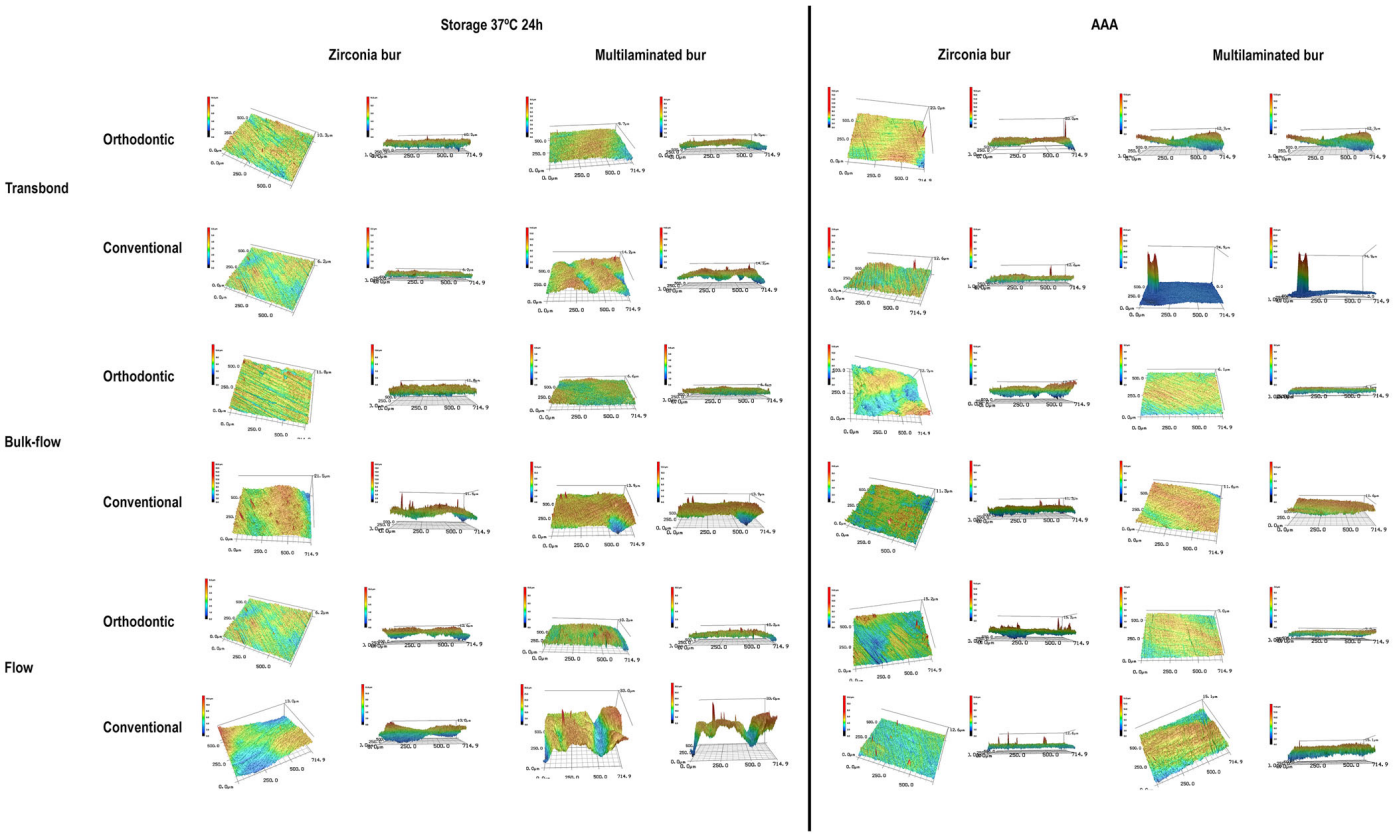
Comparative images of the enamel surface after attachment removal and final polishing for all treatment groups.

Representative confocal laser scanning microscopy images of all groups after final polishing are presented in Figure 3.

## DISCUSSION

Attachments are bonded to enamel using adhesive and composite systems. Two adhesive systems were selected based on their clinical relevance: Transbond XT (TB), considered the gold standard in orthodontics for bracket bonding [39, 40, 41], and Single Bond Universal (SB), a widely used and clinically reliable system in restorative dentistry [42, 43, 44]. Three different composites were evaluated: Transbond XT (high mechanical resistance) [40, 45, 46], a flowable composite (low viscosity and high wettability) [47, 48, 49], and a bulk‐fill flowable composite, which combines flowability with low polymerization shrinkage and greater depth of cure [50, 51, 52].

Two removal burs were selected: a 30‐blade multilaminated tungsten carbide bur—considered the gold standard for removing composite remnants [27, 53]—and a zirconia bur reinforced with glass fibers, initially developed for stain and cement removal but increasingly used for adhesive system cleanup after orthodontic debonding [27, 54]. The carbide bur removes material by mechanical cutting, creating deep parallel grooves, whereas zirconia burs act through controlled abrasion, producing smoother surfaces with less enamel damage [27, 54, 55]. Visual inspection was used to simulate realistic clinical conditions.

Based on the results, the first null hypothesis was rejected. The fact that statistically significant differences were observed only in specific combinations suggests that enamel surface alteration is not determined by isolated material properties but rather by the interaction between composite composition, adhesive chemistry, aging condition, and bur mechanics. For instance, the combination of Transbond composite with the orthodontic adhesive system demonstrated greater sensitivity to bur type after aging. This may be explained by the high filler load and quartz particle characteristics of Transbond XT, which increase mechanical rigidity and resistance to wear, potentially leading to greater stress concentration at the adhesive–enamel interface during removal. Under aging conditions, post‐curing and matrix degradation may further increase material stiffness, amplifying the abrasive effect of multilaminated carbide burs.

Conversely, the absence of statistically significant differences in several other combinations should not be interpreted as a lack of effect, but rather as evidence of comparable mechanical behavior among those material interfaces. Flowable and bulk‐fill composites, despite differences in viscosity and filler content, may present similar removal patterns when combined with specific adhesives due to their higher organic matrix proportion and lower elastic modulus. These findings suggest that enamel preservation depends on threshold interactions between material hardness, adhesion strength, and removal technique rather than on isolated material classification.

Thus, both significant and nonsignificant findings contribute to understanding the multifactorial nature of enamel alteration after attachment removal, reinforcing that material selection should consider not only bonding performance but also removal behavior and aging response.

Under the 24 h in the incubator condition, significant differences in enamel surface roughness were observed when Transbond composite was combined with SB and removed using a multilaminated bur. The SB showed higher surface roughness values, likely due to the presence of silica fillers that enhance mechanical strength and bond performance [56, 57, 58] but also increase enamel stress during removal. The inclusion of the 10‐MDP monomer, beyond BisGMA and HEMA, may further reinforce adhesion through chemical bonding with calcium [59, 60, 61].

In interpreting these findings, it is important to consider the experimental substrate used. Although bovine enamel is widely employed in laboratory research due to its availability and ease of standardization, structural and compositional differences compared to human enamel should be acknowledged. Nevertheless, bovine enamel is considered a valid and well‐established substitute for controlled in vitro studies [47, 62, 63], allowing standardized comparisons among experimental groups. Therefore, while caution is warranted when directly extrapolating these results to clinical conditions, the use of bovine enamel does not compromise the internal validity of the present study.

The type of bur had a marked influence on enamel preservation. Zirconia burs produced smoother surfaces, while multilaminated burs generated greater roughness due to their cutting action [31, 55]. Although polishing effectively reduced surface irregularities [24, 27, 64], samples treated with zirconia burs still exhibited superior smoothness, suggesting that the grooves produced by carbide burs cannot be completely leveled during finishing [28, 55].

Among composite types, the flowable one combined with the TB and removed using a multilaminated bur exhibited higher roughness. This can be attributed to the composite's higher organic matrix and lower filler content [48], as well as its greater fluidity, which increases penetration into enamel and complicates removal [65, 66, 67].

Following AAA, significant differences were also observed for Transbond composite combined with TB when removed using zirconia burs, compared with multilaminated burs, which produced higher roughness values. These findings reflect the interaction between the adhesive system composition and the aging process. The TB, composed mainly of BIS‐GMA and TEGDMA monomers and lacking filler particles [15], is more hydrophobic than SB, reducing hydrolytic degradation [68, 69, 70]. Its higher viscosity and deeper penetration into enamel prisms [65, 71, 72] result in stronger adhesion but increased enamel loss during removal.

AAA involves UV exposure, humidity, and temperature cycling, inducing physical and chemical changes in polymeric materials [73, 74]. The additional UV energy promotes post‐curing reactions, increasing the degree of conversion and mechanical stiffness [38, 73]. Although the AAA protocol [37] used in this study (300 h UV‐B cycling) has been widely adopted in laboratory research to simulate long‐term material exposure [38, 75, 76], it is important to acknowledge its inherent limitations. The equivalence of 300 h of UV‐B cycling to approximately 1 year of clinical use is based on previously reported aging models in dental materials research, and such protocols have been used to compare relative performance between experimental groups. However, unlike the multifactorial oral environment, AAA does not fully replicate intraoral conditions, including continuous saliva exposure, pH fluctuations, enzymatic activity, and masticatory loading. Therefore, while this protocol provides a standardized method for material aging, caution should be exercised when extrapolating these findings directly to clinical scenarios.

Transbond XT, containing 70%–80% quartz fillers [15, 77, 78], exhibits excellent bond strength but may be more aggressive to enamel during debonding due to its hardness and filler particle size [45, 79, 80]. Thermal stress during aging preferentially degrades the organic matrix, exposing large quartz particles and increasing surface roughness [36, 81]. The larger and more angular these particles are, the greater the enamel wear caused during removal [82].

The combined degradation of both the resin and the adhesive system interface explains the cumulative surface damage, as both components undergo post‐curing and aging. Values of surface roughness above 0.2 µm favor biofilm retention and increase caries risk, whereas values above 0.3 µm can be detected by the tongue and cause discomfort [81, 83]. Confocal laser scanning microscopy analysis confirmed that even after polishing, the enamel surface did not recover its original smoothness or morphology, leading to the rejection of the second null hypothesis. Surface irregularities lead to surface roughness and they are created during the removal. The finishing and polishing procedure can soften the resin matrix due to the production of heat as well as by dislodgement or debonding of fillers [84], leaving scratch lines and cracks on the surface.

Despite the controlled conditions adopted, the findings of this study should be interpreted in light of some limitations. The in vitro design does not fully replicate the biological and mechanical complexity of the oral environment, and the absence of magnification during attachment removal may have reduced visual control and precision during the procedure. In addition, operator proficiency is a factor that may influence the outcomes. Consequently, caution is warranted when extrapolating these results to clinical practice, and further well‐designed clinical studies are necessary to confirm and expand upon the present findings.

Overall, attachment removal is an irreversible process that inevitably alters the enamel surface, with outcomes strongly dependent on the operator's technique and the combination of materials used. In future studies, it would be important to standardize the use of magnification during resin removal procedures, to evaluate the specific use of zirconia burs, to assess the wear of these composite materials during aligner use, and to investigate the bond strength of them when used with the tested adhesives. Additionally, in situ studies should be conducted to incorporate clinical oral conditions and better simulate the intraoral environment.

## CONCLUSION

Within the limitations of this current in vitro study, it was concluded that:

1. Enamel surface roughness was significantly affected after attachment removal, rejecting the null hypothesis. These changes occurred regardless of the composite resin, adhesive system, or bur employed, indicating that all tested combinations produced measurable alterations to enamel topography.
2. Enamel morphology was also significantly altered following attachment removal, rejecting the second null hypothesis. None of the protocols—whether differing in resin type, adhesive system, or bur—were capable of fully preserving the original enamel structure.
3. These findings emphasize the need to consider not only the bonding performance of materials used for attachment fabrication but also their interaction with removal techniques and post‐treatment clinical conditions. Final polishing remains essential and should be systematically incorporated to minimize surface damage and improve enamel smoothness after debonding.

## AUTHOR CONTRIBUTIONS

**Conceeptualization**: Marilia Daniela Busnardo Canadas Canadas, Fernanda de Carvalho Panzeri. **Methodology**: Marilia Daniela Busnardo Canadas. **Investigation**: Marilia Daniela Busnardo Canadas, Ana Beatriz Silva Sousa. **Resources**: Marilia Daniela Busnardo Canadas. **Writing—original draft**: Ana Beatriz Silva Sousa. **Formal analysis**: Fernanda de Carvalho Panzeri. **Supervision**: Fernanda de Carvalho Panzeri. **Writing—review and editing**: Fernanda de Carvalho Panzeri.

## CONFLICT OF INTEREST STATEMENT

The authors declare no conflicts of interest.
